# An Expanded Conceptual Framework for Understanding Irritability in Childhood: The Role of Cognitive Control Processes

**DOI:** 10.1007/s10567-024-00489-0

**Published:** 2024-06-10

**Authors:** Olivia M. Elvin, Kathryn L. Modecki, Allison M. Waters

**Affiliations:** 1 School of Applied Psychology, Griffith University, Mount Gravatt Campus, Brisbane, QLD Australia; 2 Centre for Mental Health and School of Applied Psychology, Griffith University, Mount Gravatt Campus, Brisbane, QLD Australia; 3 School of Psychological Science, University of Western Australia & Telethon Kids Institute, Perth, Australia

**Keywords:** Irritability, Cognitive control, Setting factors, Expanded conceptual framework, Middle childhood

## Abstract

Children prone to irritability experience significant functional impairments and internalising and externalising problems. Contemporary models have sought to elucidate the underlying mechanisms in irritability, such as aberrant threat and reward biases to improve interventions. However, the cognitive control processes that underlie threat (e.g., attention towards threats) and reward (e.g., attention towards reward-related cues) biases and the factors which influence the differential activation of positive and negative valence systems and thus leading to maladaptive activation of cognitive control processes (i.e., proactive and reactive control) are unclear. Thus, we aim to integrate extant theoretical and empirical research to elucidate the cognitive control processes underlying threat and reward processing that contribute to irritability in middle childhood and provide a guiding framework for future research and treatment. We propose an expanded conceptual framework of irritability that includes broad intraindividual and environmental vulnerability factors and propose proximal ‘setting’ factors that activate the negative valence and positive valence systems and proactive and reactive cognitive control processes which underpin the expression and progression of irritability. We consider the implications of this expanded conceptualisation of irritability and provide suggestions for future research.

## Introduction and Background

Irritability is not only associated with poorer functioning concurrently (Beauchaine & Cicchetti, 2019; Elvin et al., 2021), but also predicts poorer longitudinal outcomes (i.e., development of psychopathology; Copeland et al., 2015; Vidal-Ribas et al., 2016). Given that irritability is one of the most common reasons youth are referred for psychiatric care (Collishaw et al., 2010; Evans et al., 2022; Stringaris, 2011), researchers are increasingly seeking to understand the mechanisms that underlie irritability (Brotman et al., 2017a, 2017b) and elucidate key targets for intervention (Kircanski et al., 2019). First, we review current definitions and transdiagnostic perspectives of irritability. Following this, we present the proposed expanded conceptual framework and integrate the literature in relation to the key tenets in the irritability, including (A) the environmental and intraindividual vulnerability factors implicated in youth with heightened irritability; (B) the ‘setting’ factors that have a bearing on the expression and phenomenology of irritability; (C) the differential pathways of positive and negative system activation in line with the RDoC perspective; (D) the underlying cognitive control processes that are maladaptively activated under threat and reward conditions; and (E) the varying outcomes of the expression of irritability and its longitudinal course. Finally, we explore the implications of this framework for theory and intervention and provide directions for future research.

### Irritability Defined

Irritability is a diagnostic characteristic spanning across both internalising and externalising disorders (American Psychological Association, 2000). Irritability has been defined as a trait (Beauchaine & Tackett, 2019), falling on a spectrum alongside related phenotypes of reactive aggression (considered a severe externalised presentation of irritability; Leibenluft, 2017) and anger, within the broader syndrome of negative affect (Stringaris et al., 2017; Vidal-Ribas et al., 2016). It is characterised by a proneness to experience anger and aggression (Barata et al., 2016; Toohey & DiGiuseppe, 2017) and can be understood as two distinct, but interrelated constructs known as tonic and phasic irritability. Here, tonic irritability is defined as persistently angry, grumpy or grouchy mood, whereas phasic irritability refers to discrete episodes of excessive temper or frustration such as crying, shouting or stomping (i.e., temper outburst) which may also include violence toward people or objects (i.e., temper tantrum) (Copeland et al., 2015). Notably, although the DSM-5 has recognised these two components of irritability as separate symptoms in the context of disorders such as Oppositional Defiant Disorder (ODD) and Disruptive Mood Dysregulation Disorder (DMDD), and recent strides have been taken to differentiate tonic from phasic forms of irritability (Hirsch et al., 2021; Vidal-Ribas & Stringaris, 2021), to date much of the literature has measured irritability as a unitary construct (i.e., via the ARI; Stringaris et al., 2012a, 2012b) or as a symptom in the context of other disorders that have irritability as an associated feature, including depression (Vidal-Ribas & Stringaris, 2021), anxiety (Brotman et al., 2017a, 2017b; Comer et al., 2012; Rappaport et al., 2018) and Attention-Deficit/Hyperactivity Disorder (ADHD) (Benarous et al., 2017; Eyre et al., 2017; Mulraney et al., 2016; Waxmonsky et al., 2017).

### Irritability in Clinical Populations

DMDD was introduced into the DSM-5 given that a subset of youth display severe and impairing levels of irritability by elementary school, occurring in approximately 0.8 to 2.8% of school aged youth (Baweja et al., 2016; Copeland et al., 2013). Although some prior studies have raised concerns about DMDD as a disorder in its own right given its high comorbidity rates with both emotional and disruptive disorders (estimated at 62–92%; Copeland et al., 2013), it is clear there are long-term impacts of DMDD into adulthood (Copeland et al., 2014). As a key feature of childhood DMDD, irritability is also present and potentially impairing within community samples of youth who do not meet full criteria for DMDD, with prevalence estimates ranging from seven (Baweja et al., 2016; Moore et al., 2019) to 57% (Copeland et al., 2015). In Karlovich et al. (2023), the lifetime prevalence of irritability was estimated to be as high as 79.5% in a large epidemiological study of adolescents. Irritability has been found to have longitudinal associations with internalising psychopathology (e.g. anxiety, depression; Copeland et al., 2015), externalising psychopathology (e.g. ODD; Dougherty et al., 2015) and increased risk for suicidal behaviour (Benarous et al., 2019). Moreover, irritability has been associated with more general poor functioning in youth, including peer problems (Beauchaine & Cicchetti, 2019; Elvin et al., 2021) and high rates of school suspensions and family disruptions (Copeland et al., 2015).

### Course and Prevalence of Irritability

Although transient irritability is common in youth and particularly in preschool-aged children (Wakschlag et al., 2015), it also appears to undergo developmental change across the course of childhood. For example, longitudinal research has described developmental trajectories through middle childhood (i.e., Orri et al., 2019; Pagliaccio et al., 2018; Wiggins et al., 2014). While the specific nature of individual trajectories differs, each describes a majority of young people who exhibit stable low or decreasing irritability symptoms in line with maturation (i.e., 60.8% of children in the Fragile Families study demonstrated low and decreasing levels of irritability; Wiggins et al., 2014). Examination of longitudinal trajectories highlight both a moderately high and stable group of irritability in addition to a developmentally escalating group across both a high risk (Wiggins et al., 2014) and normative (Orri et al., 2019) sample of children, pointing to the likelihood that at-risk children may either maintain initially high levels of irritability or may present as initially low or moderate, but then escalate in counter to developmental norms. Indeed, decreases in irritability likely correspond to increasing ability to regulate and appropriately express emotions, allowing for normative declines with age (Stringaris & Taylor, 2015; Wakschlag et al., 2018). However, stable high and increasing irritability groups represent particularly concerning trajectories, given they developed counter to normative maturational changes in self-regulation, and these young people arguably represent those most likely to present for treatment. These differing trajectories of irritability highlight that among both normative and at-risk samples, children exhibiting high levels of irritability can be identified during the preschool and middle childhood years, through the process of equifinality. At the same time, early irritability also appears to relate to multifinality in that it is transdiagnostic, relating to both internalising and externalising symptomology (Finlay-Jones et al., 2023; Pagliaccio et al., 2018).

## An Expanded Conceptual Framework of Irritability in Childhood

While childhood irritability is relatively common and can be associated with poor concurrent and longitudinal outcomes, only recently have researchers attempted to understand the processes underlying the development of irritability (i.e., Brotman et al., 2017a, 2017b). Alongside more recent adaptations (Kircanski et al., 2019), aberrant threat and reward processing have been specified as key psychological mechanisms within these models. Indeed, the translational model of irritability proposed by Brotman et al., (2017a, 2017b) first provided insights into the components of threat and reward processing that interact and underlie childhood irritability. Kircanski et al. (2019) then included inhibitory control in their treatment model, providing further insight into factors thought to underlie irritability. Some studies (Brænden et al., 2022; Myers et al., 2017) have also explored irritability in the context of the Research Domain Criteria (RDoC) perspective (Insel et al., 2010; NIMH, 2021). Although this prior research has allowed for progression in understanding of the mechanisms involved in irritability and potential treatment targets, gaps critical for future treatment models remain. In particular, the cognitive control processes that underlie threat and reward biases, the proximal conditions that ‘set the scene’ and trigger the activation of these processes underlying threat and reward biases, and how they may be linked to differences in the expression (i.e., tonic and/or phasic) and progression (i.e., to DMDD or co-occurring psychopathology) of irritability remains poorly understood. Further, there are key differential pathways of RDoC negative and positive valence systems, that once activated appear to play a pivotal role in the ability of youth to flexibly recruit cognitive (i.e., proactive and reactive) control. Integrating these perspectives, our review aims to explore the cognitive processes that underlie and maintain the mechanisms of threat and reward processing in childhood irritability. In particular, we build on past frameworks to examine both tonic and phasic expressions of irritability and the processes that underlie threat and reward processing through the application of existing cognitive control theories (i.e., Braver, 2012) and the RDoC domains. As cognitive control develops over the first ten years of life (Munakata et al., 2012; Troller-Renfree et al., 2020), this review aims to focus on irritability within middle childhood, however studies examining samples of youth within early childhood over a longitudinal course can also provide further understanding about how these mechanisms may arise and develop over time. Further, although irritability does present in the context of many disorders (i.e., ODD), here we focus specifically on studies with irritability as an individually measured construct or in the context of disorders with irritability as its key feature (i.e., DMDD). To this end, we aim to shed further light on childhood irritability via a proposed framework which integrates prior theoretical and empirical research and seeks to guide future research and treatment.

### Setting the Context for the Expanded Conceptual Framework: Pathways of System Activation

Important in these accounts of the processes that may underlie the systems and processes in irritability is in understanding the role of dimensional models, such as that of the RDoC perspective (Insel et al., 2010; NIMH, 2021). Given the challenges characterising irritability and DMDD, some shifts in understanding irritability have included the application of the RDoC criteria (i.e., Brænden et al., 2022; Meyers et al., 2017). Although irritability cuts across many RDoC domains, with some prior studies drawing upon some aspects of the RDoC matrix (i.e., Brænden et al.,’s, 2022), here we focus on three key systems and specific constructs within these domains (NIMH, 2021). First, *negative valence systems* underlie responses to aversive situations or contexts, likely arising from the activation of real or perceived threat and the withdrawal or prevention of reward (i.e., frustrative nonreward). Second, *positive valence systems* underlie responses to motivational situations or context, such as reward, and include responsiveness to received or anticipated rewards, reward valuation (i.e., under or over valuation of possible rewards), and reward learning (i.e., predicting rewards). Third, *cognitive systems* are responsible for a range of cognitive processes, including cognitive control, which refers to the modulation of cognitive and emotional systems for the purposes of goal-directed behaviour. Although irritability may be understood as arising from sensitivity to maladaptive negative and positive valence system activation, with cognitive control linking across these varying domains, the specific processes that underlie cognitive control remains unclear. Indeed, although much research alludes to deficits in cognitive control (i.e., inhibitory control deficits; Brotman et al., 2017a, 2017b), the explicit cognitive control processes underlying these differences in threat and reward biases requires further research. Further, given prior studies (i.e., Nigg, 2017) have highlighted the range of constructs and terms used to refer to concepts such as cognitive control, here we focus on the theoretical application of a specific framework of cognitive control and propose its application in conceptualising irritability. Thus, here we apply the Dual Mechanism of Control (DMC) framework proposed by Braver (2012), which draws together the discrete but interacting cognitive processes of *proactive control* (i.e., actively selecting goal-relevant information and filtering out goal-irrelevant information) and *reactive control* (i.e., self-correcting responses following interference in a goal-directed task) (Braver, 2012; Braver et al., 2009).

**Fig. 1:**
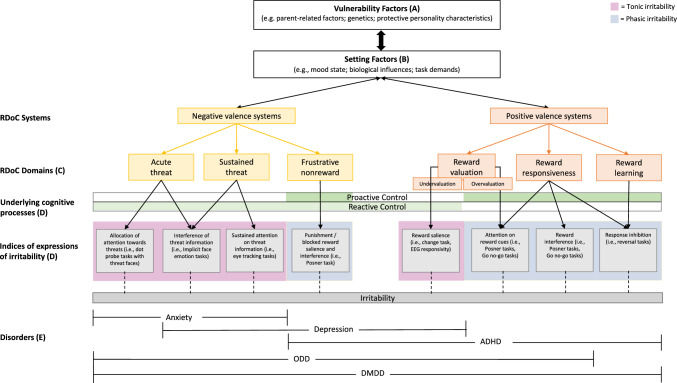
Expanded and conceptual framework of the progression and expression of tonic and phasic irritability linked to underlying system activations, cognitive control processes, and psychopathology

As a result, and presented in Fig. 1, we conceptualise irritability with respect to the (A) vulnerability factors, (B) setting factors, (C) differential pathways of positive and negative valence system activation, (D) underlying cognitive processes that cut across these negative and positive valence systems, and (E) expression and progression over the longer term with respect to associated diagnoses. This expanded conceptual framework draws together the evidence regarding vulnerability factors for irritability and suggests that particular proximal setting conditions (i.e., greater environmental demands, negative mood) in which vulnerability factors may be more impactful (i.e., low SES, harsh parenting) are likely to activate positive and negative valence systems (Fig. 1C) and accentuate maladaptive activation of cognitive control (i.e., Fig. 1D), thus contributing to the expression, and differential outcomes of, irritability in youth. We further highlight how tonic and phasic expressions of irritability may be associated with differential patterns of activation in youth and are thus more closely associated with certain presentations of youth psychopathology in the longer term. Illustratively, a history of greater exposure to harsh parenting (vulnerability factors- environmental factors; Fig. 1A) in addition to child temperamental traits of high negative affect (vulnerability factors- intrapersonal factors; Fig. 1A) may interact to increase a child’s vulnerability to experiencing irritability. In any given situation, this child may be impacted by various setting factors (i.e., feeling tired, increasing task demands; Fig. 1B) which triggers activation of the negative and positive valence systems (Fig. 1C), to which underlying cognitive control processes are activated maladaptively (Fig. 1D) and rather than downregulating negative states, increases the expression and intensity of irritability. The differential activation of processes underlying negative and positive systems with the experience of these proximal events associated with the maladaptive activation of the cognitive control systems (which underlies expression of threat and reward biases observed in prior studies) highlights the expression and progression of irritability and is outlined in Fig. 1. Indeed, using a broad lens, irritability appears equifinal, however there are specific pathways that appear to intersect, leading to differences in the expression of irritability and the processes that in the longer term are observed in the context of psychopathology. Outlined below are the pathways of system activations as viewed in the context of the RDoC criteria (Insel et al., 2010) and proposed to differentially impact the recruitment of flexible cognitive (proactive and reactive) control processes in the service of goal-directed tasks in irritable youth.

1. *Negative valence system overactivation*. Negative valence systems are overactivated via threat systems, such as perceived or actual acute threat (i.e., making an error on a task; disagreement with a peer) and/or sustained threat (i.e., ongoing peer conflict, ongoing academic demands). Once the negative valence system is activated, the capacity for employment of proactive control is impacted through greater load on cognitive capacity (i.e., working memory) and/or the impacts of reduced motivation to perform and adhere to task goals, leading to an overreliance on reactive control. In the short-term, this pathway is likely to be expressed as tonic irritability (i.e., irritable mood). Over the longer term, experiences of negative valence system overactivation likely progresses into presentations of both internalising presentations (i.e., increased negative mood; increased hypervigilance) such as anxiety and depression, and externalising presentations (i.e., increased negative mood; increased peer conflict), such as ODD.
2. *Frustrative nonreward.* Overactivation of the negative valence system may also occur through incongruence between positive and negative expectancies, including the withdrawal or prevention of a reward (i.e., placing second in a sporting event or academic task in which the child expects to win or perform well). This frustrative nonreward can be observed during goal-directed tasks, in which a child may initially employ proactive control inflexibly, impacting on their performance (i.e., attempting to speed up to perform faster and falling over, or making more errors), and failing to move between proactive control and reactive control as required (i.e., becoming distracted by a peer being ahead and thus having difficulty focusing attention on their own performance). This pattern of activation may lead to greater phasic expressions of irritability (i.e., outbursts) due to the lack of perceived or actual reward. Over the longer term, sustained non-reward (real and perceived) may lead to depression (i.e., decreased motivation for tasks) and/or externalising presentations of ADHD or ODD (i.e., increased irritable outbursts and non-compliance with nonpreferred tasks).
3. *Positive valence system underactivation.* Underactivation of the positive valence system may arise through undervaluation of potential rewards (i.e., opportunities for enjoyable/preferred activities not forthcoming; having to complete a task with high effort for little or no reward). Such underactivation of the positive valence system may reduce the likelihood of a child engaging in more cognitively demanding tasks (i.e., proactive control) to gain reward and thus an overreliance on reactive control, and poorer task performance. In the short-term, this pathway may increase tonic expressions of irritability, with ongoing impacts on a low positive mood and other features of depression, such as lack of motivation or enjoyable activities and/or ADHD (i.e., reduced task engagement and/or completion).
4. *Positive valence system overactivation.* Overactivation of positive valence systems may arise from the overvaluation of rewards (i.e., increased motivation due to the salience of incentives), reward responsiveness (i.e., focusing on the total score or increasing rewards when competing), and reward learning (i.e., reward prediction errors, such as predicting or anticipating receiving a reward; habit, such as challenges with response inhibition in response to previously learned responding). This overactivation of the positive valence system is likely to enhance a child’s motivation to employ cognitively demanding processes (such as proactive control) in order to receive the reward. However, inflexible proactive control in high-reward situations may trigger frustrative nonreward in situations in which high reward expectancies do not eventuate (i.e., reward prediction errors), resulting in increased phasic expressions of irritability and impacting longer-term interactions with peers. This pathway is likely to be expressed as phasic, impulsive behaviours characteristic of ADHD (predominantly hyperactive/impulsive type) in which high reward drive is common (i.e., increased competitiveness with peers, increased social conflict).

Although the expression of positive and negative valence systems as patterns of underactivation, overactivation, or frustrative nonreward (i.e., incongruence) occurs in various presentations of internalising and externalising psychopathology in which irritability is a core component, the combination of all four patterns of activation outlined above are proposed to occur in the context of DMDD. Although initially youth with DMDD may present with one or more of these aberrant patterns of systems activation, it is likely that over the longer-term, the activation of these patterns, and the associated detrimental outcomes, becomes more chronic and severe, leading to more frequent outbursts and persistently irritable mood which in turn further triggers maladaptive positive and negative valence system activation in a vicious perpetuating cycle. Taken together, this conceptual framework helps to explain nuanced differences in findings on threat and reward processing biases and the varied outcomes observed among irritable youth. It also explicates further treatment targets and approaches for childhood irritability, such as developing or enhancing treatments that more effectively target these underlying patterns of system activation.

## Vulnerability Factors (A)

Presented in Fig. 1A, the model highlights vulnerability factors thought to increase a child’s susceptibility to experiencing irritability. Similar to prior studies (Brotman et al., 2017a, 2017b; Kircanski et al., 2018), we have highlighted evidence specific to vulnerabilities for irritability, however we also draw upon transdiagnostic vulnerability factors common across many childhood psychopathologies given that irritability presents across the spectrum of externalising and internalising disorders (i.e., American Psychological Association, 2000). Such factors include a combination of environmental factors (e.g., parent factors), and intrapersonal factors (e.g., genetics, protective personality characteristics) thought to predispose youth to experiencing irritability.

### Environmental Factors

Some mixed evidence has been found on the direct relationship between parent factors (i.e., parent psychopathology, parent–child relationships) and irritability over the longer term. Prior findings have found associations with family history of suicidality (Krieger et al., 2013), depression (Dougherty et al., 2013; Krieger et al., 2013; Propper et al., 2017; Sorcher et al., 2022), and parent irritability (Di Giunta et al., 2020) with childhood irritability. Studies have implicated higher rates of family dysfunction (i.e., higher family conflict, poorer communication) in youth with severe irritability (Zendarski et al., 2023) in addition to associations between childhood irritability and exposure to violence in mothers (Wiggins et al., 2014). Alongside parent psychopathology, harsh parenting has been linked to more severe irritability (Wiggins et al., 2014) and its related phenotypes (i.e., reactive aggression; Vitaro et al., 2006). Social learning also plays a large role in the development of child outcomes, with family experiences altering a child’s expectations of rewards and punishments (Cairns & Cairns, 2006) through instrumental learning. Deficits in instrumental learning, a form of operant learning defined as the likelihood of a behaviour being strengthened or weakened by reward and punishment, is also thought to play a role in the development of irritability (McSweeney & Murphy, 2014). These learned deficits may be maintained across child development through parental responding and modelling, impacting the stable and predictable provision of appropriate responding to desirable and undesirable behaviour (Brotman et al., 2017a, 2017b; Brotman et al., 2017a, 2017b) and thus influencing a child’s expectancies in various settings. Thus, these findings highlight the influence of parent and family as environmental vulnerability factors which play a role in the onset and maintenance of childhood irritability.

Alongside and related to parent-factors, socioeconomic status (SES) (Davis et al., 2010; Reiss et al., 2019) and exposure to traumatic life events (i.e., exposure to violence; Briggs-Gowan et al., 2012) have been linked with many adverse child outcomes, such as the onset of mental health problems. In the study conducted by Wiggins et al. (2014), more severe trajectories of irritability were associated with environmental factors, including lower material educational attainment and worse neighbourhoods. Indeed, exposure to stressful life events has been associated with increased anger and irritability symptoms in youth, alongside higher rates of internalising symptomology and externalising problems (i.e., school dropout, drug use) (Roberts et al., 2018). Further, given that irritability is a symptom of PTSD (American Psychological Association, 2000), exposure to traumatic or stressful life events in childhood appears to predispose some youth to experiencing irritability in childhood and later life. Together, environmental factors such as parent-related factors, SES and exposure to trauma may increase youth vulnerability to experiencing irritability, particularly when combined with intrapersonal vulnerability factors.

### Intrapersonal Factors

*Genetics*. Genetic and environmental influences are thought to play parallel roles in the development of irritability in children (Coccaro et al., 1997). Genetic factors are associated with the development of irritability, with males having stable genetic influences in early childhood through to young adulthood. Comparatively, irritability in females is influenced by genetic factors early in development, which decrease with time (Roberson-Nay et al., 2015). The heritability of irritability is moderate (Vidal-Ribas et al., 2016), reported as approximately 0.4–0.6 (Savage et al., 2015; Stringaris et al., 2012a, 2012b) and associated with genetic risk for anxiety and depression (Rappaport et al., 2018; Stringaris et al., 2012a, 2012b; Vidal-Ribas et al., 2016). On the other hand, irritability has also been found to share moderate genetic risk factors with ADHD in young children decreasing with age, demonstrating shared risk across the internalising and externalising spectrum. In the case of DMDD, although this has been estimated to be moderately heritable (59% heritability), it was found to relate more closely to phasic irritability (61–63% heritability), rather than to tonic irritability (54% heritability) which was less stable over time (Moore et al., 2019). Thus, irritability appears to be moderately heritable in childhood, with some shared genetic risk markers for the development of psychopathology.

*Neurobiological mechanisms*. Intrapersonal factors that may influence a child’s susceptibility to irritability may also include underlying brain structure and function (Brotman et al., 2017a, 2017b; Perlman et al., 2015; Toohey & DiGiuseppe, 2017). Prior research has indicated that brain function develops alongside brain structure, integrating complex networks (i.e., cognitive control; Luna et al., 2015). Differences in neural activation have been found in studies comparing irritable and non-irritable youth (e.g., Adleman et al., 2011; Liuzzi et al., 2023; Perlman et al., 2014; Rich et al., 2007). Functional connectivity within irritability is thought to vary as a result of development across the lifespan and implicates the involvement of multiple functional networks (Nielsen et al., 2021). Specifically, findings have suggested greater frontal-striatal activation in the dorsolateral prefrontal cortex, inferior frontal gyrus, caudate, and anterior cingulate cortex in irritable youth (Tseng et al., 2018) in addition to changes in activation patters in the middle frontal gyrus and parietal cortex (Deveney et al., 2013; Perlman et al., 2015). Some findings also exist in relation to underlying differences in brain structure compared to function. Specifically, Cardinale et al. (2019) suggested functional rather than structural differences in neurodevelopment in a sample of 331 clinically irritable youth. These findings were similar to prior research investigating brain abnormalities within populations with high irritability, leading to suggestions that irritability is associated with functional rather than structural differences in neurology (Adleman et al., 2012). On the other hand, irritability has been linked with structural abnormalities in the superior frontal gyrus (associated with attention control and response inhibition), with some findings suggesting alterations in grey matter and cortical thickness in irritable youth (Gold et al., 2016; Pagliaccio et al., 2018). Jirsaraie et al. (2019) found cortical thinning in brain regions implicated in emotion regulation and identification in a sample of 137 clinically irritable youth aged 8- to 22-years-old. Similarly, structural abnormalities were found in a sample of 10- to 18-year-olds with severe irritability relative to healthy controls, including decreased cortical thickness, grey matter volume, and local gyrification index in the superior frontal gyrus (Seok et al., 2021). Although findings point to more consistent functional neural differences in irritability, further research is needed to determine structural brain differences in these youth given mixed findings.

*Temperament*. Child temperament is thought to be a collection of enduring traits that may put children at risk of developing psychopathology (Nigg, 2006). Illustrating this, high reactive (i.e., emotionality) and low regulatory (i.e., effortful control) temperamental traits have been associated with psychological problems (Muris & Ollendick, 2005). Indeed, in a sample of 6-year-olds both tonic and phasic irritability were associated with parent reporting of temperamental traits, including negative affect, with phasic irritability also linked to high surgency/approach motivation and low effortful control (Silver et al., 2023a, 2023b). Further, young children with greater parent-reported temperamental surgency or extraversion demonstrate longitudinal associations with greater frustration and anger at 5 to 6 years of age (Berdan et al., 2008). For example, within a preschool sample (Nozadi et al., 2018), low effortful control (comprised of self-regulation of emotion, behaviour, and cognition) was found to condition the relation between an angry temperament and longer durations of looking at angry faces on an eye tracking task. This suggests that reduced effortful control in younger children predisposes youth with an angry temperament to develop biases in attention towards threat. However, other research has suggested that children with a difficult (i.e., under controlled) temperament are not rated differently based on their externalising problems by age 7 (Miner & Clarke-Stewart, 2008). Ezpeleta et al. (2019) found that irritability and parenting mediate the relation from temperament to psychopathology, suggesting that a child’s temperament may predispose some youth to experiencing irritability. These approaches in turn are likely to exacerbate negative qualities in children, such as irritability.

*Protective personality characteristics*. Although non-normative trajectories of irritability (see Orri et al., 2019; Pagliaccio et al., 2018; Wiggins et al., 2014) are generally reflective of patterns of stable or increasing irritability across childhood, some children demonstrate severe levels of irritability at a young age, which decline to close to normative levels by middle childhood (Orri et al., 2019; Wiggins et al., 2014). Given these declines, positive wellbeing characteristics (i.e., child resilience or hope) may explain some differences in these trajectories, despite being exposed to factors such as mothers with a history of depression or trauma that should place them at elevated risk (Wiggins et al., 2014). Although studies specific to positive wellbeing characteristics and irritability are scarce, recent evidence suggests that higher self-regulation of negative emotion, hope and flourishing appear to be inter-related with lower levels of irritability within youth (Elvin et al., 2021). This suggests that some positive wellbeing characteristics may be protective for some children, and that low levels of these characteristics may put some children at risk of higher irritability. However, further research is still required to understand the role of positive wellbeing characteristics in youth with high levels of irritability and how this may relate specifically to setting factors (Fig. 1B) and underlying patterns of system activation (Fig. 1C) and cognitive processes (Fig. 1D).

*Sensory differences*. Although primarily observed within neurodevelopmentally diverse populations (i.e., Autism Spectrum Disorders, ADHD; Little et al., 2018), sensory processing differences (i.e., difficulty regulating sensory information such as touch, sound, taste) have also been found across internalising and externalising disorders (Gourley et al., 2013). Irritability appears to be linked to greater sensitivity to the environment (e.g., hypersensitivity to noise; Toohey & DiGiuseppe, 2017), and within a sample of 6- to 16-year-olds, those with DMDD had a significantly higher number of sensory processing difficulties, and in particular, more difficulty modulating environmental sensory input relative to youth with other emotional disorders (Benarous et al., 2020). Thus, it is likely that these sensory sensitivities in youth with heightened irritability interact with specific setting factors (e.g., increased environmental input) which in turn influences their mood and capacity to flexibly recruit cognitive control, particularly when acutely frustrated.

Importantly, vulnerability factors are proposed to interact with setting factors (Fig. 1B and below), differentially activating negative and positive valence systems (Fig. 1C) and impacting the recruitment of cognitive control resources (i.e., proactive and reactive control; Fig. 1D) and the expression and progression of irritability (Fig. 1E).

## Setting Factors (B)

Setting factors (Fig. 1B), i.e., contextual or immediate precursors that ‘set the scene’ for engagement of underlying processes have not been explicitly outlined in prior models of irritability. However, some researchers have considered conditions under which threat and reward biases thought to underlie irritability may be intensified, such as when irritable youth are acutely angry or frustrated (i.e., Deveney et al., 2013; He et al., 2013). The setting factors proposed here are interrelated circumstances which appear to exacerbate or ‘activate’ the positive and negative valence systems, leading to maladaptive employment of cognitive control and the expression of irritability. Setting factors (Fig. 1B) include internal factors (i.e., current mood) and environmental demands (i.e., task requirements) that may influence outcomes alone or in amalgamation, in activating situations (e.g., within the classroom, playing with peers) and/or resulting from changes in internal states (e.g., as a child becomes more tired or hungry).

### Internal Factors

*Biological influences*. Internal factors that may directly or indirectly influence irritability include biological factors, such as lack of sleep, pain, and hunger (Toohey & DiGiuseppe, 2017). Indeed, emotion dysregulation has been associated with poor sleep, whereby youth who experienced greater sleep loss were more reactive to negative environmental stimuli (Goldschmied, 2019). Further, in a sample of 613 youth, poorer sleep efficiency and shorter sleep duration was associated with greater temperamental anger/frustration in middle childhood (Miadich et al., 2020). Although sleep problems have been found to be associated with DMDD, with higher instances of sleep difficulty in youth with higher irritability, sleep problems are linked to shared relationships with other disorders as well (i.e., ADHD), rather than with the onset of DMDD specifically (Waxmonsky et al., 2017). Findings therefore point to ongoing sleep problems likely contributing to greater irritability and susceptibility to negative environmental factors via likely activation of the negative valence system.

*Mood state*. Likely associated with biological influences, a child’s internal mood state appears to impact on their ability to engage effectively in a goal-directed activity. Disorders that impact on an individual’s resources (i.e., excessive worrying impacting on sleep and energy; anhedonia impacting on motivation) likely further contribute to irritability due to the changes in biological factors associated with attempts to manage symptoms (thus reducing working memory capacity) (Toohey & DiGiuseppe, 2017). Moreover, a child’s mood at the time can also influence the ability to engage cognitive processes and regulate irritability. As seen in the poorer performances in reward tasks following mood induction or frustration (Deveney et al., 2013; He et al., 2013), an individual’s mood when completing a task is an important consideration for threat processing as well. Despite differences in performance that have been observed for irritable compared to non-irritable youth under neutral conditions (e.g., Dickstein et al., 2007), higher levels of frustration impacting on task performance (Deveney et al., 2013; He et al., 2013; Tseng et al., 2018) certainly underscores the need to further account for the impacts of a child’s mood on task performance (i.e., negative valence system activation). On the other hand, given that some youths appear to respond more positively to the presence of rewards (i.e., in Kessel et al., 2016a, 2016b; Perlman et al., 2015), it is likely that positive mood (i.e., positive valence system activation), which may be elicited resulting from the possibility of receiving a reward, may enhance task performance in youth prone to irritability when not acutely frustrated or irritable. Thus, alongside transient factors such as sleep and hunger, a child’s current mood also appears to impact on irritability and their capacity to engage with a task.

### Environmental Demands

*Task demands*. In addition to internal factors and expectancies, environmental demands also appear to ‘set the scene’ for underlying system activation and irritability in youth. Specifically, tasks with increasing complexity (Adleman et al., 2011; Deveney et al., 2013; Li et al., 2023) or length (Liuzzi et al., 2020) appear to have a greater negative impact on those with high versus low levels of irritability. More challenging task demands also likely interact with a child’s negative mood state, thereby adversely influencing negative and positive valence systems and cognitive control activation underlying threat and reward processing and thus, increased irritability.

## Integrating Vulnerability (A) and Setting Factors (B)

Although this review provides an account of the research to-date of factors thought to increase an individual’s vulnerability to experiencing irritability (‘vulnerability factors’; Fig. 1A) and the more immediate contextual factors that may activate or exacerbate maladaptive responding (‘setting factors’; Fig. 1B), these factors are also thought to be interrelated. Indeed, although some studies refer to irritability as an underlying disposition or ‘trait’ (i.e., Beauchaine & Tackett, 2019), other studies have positioned irritability as a state-like concept arising from momentary experiences (Toohey & Digiuseppe, 2017). More recently, irritability has been described as a distinct factor arising from early temperamental vulnerabilities that is multifaceted and influenced across development (i.e., Silver et al., 2023a, 2023b; Sorcher et al., 2024). Indeed, the combination of specific vulnerability factors in circumstances or ‘settings’ which may lead to activation of positive and negative valence systems may then exacerbate a child’s predisposition to experiencing irritability. For example, although harsh parenting was highlighted as a vulnerability factor (Wiggins et al., 2014), children with more severe irritability appear to evoke harsher and more hostile parenting styles, indicating a bidirectional relationship (Hakulinen et al., 2013; Kiff et al., 2011). As such, although negative parenting (i.e., shouting and smacking) has been found to increase irritability in middle childhood, negative parenting also appears to be driven by greater irritability in young childhood (i.e., 4 years of age; Oliver, 2015). Together, the bidirectional influences of child temperament and parenting appear to interact to influence child outcomes and adjustment. Moreover, Lengua (2006) suggested that irritable and difficult to manage temperamental traits in children engender frustration in parents and thus lead to more inconsistent parenting approaches (and in turn promoting incompatible setting conditions; Fig. 1B), which may for example lead to changes in parental reward contingencies and foster negative mood states. Thus, although we have outlined both some key vulnerability factors and setting factors implicated in childhood irritability, it is important to note these factors are interrelated, as depicted by the bidirectional arrow in Fig. 1. Early vulnerability factors and setting factors also appear to be interrelated in the case of both threat and reward expectancies in irritable youth.

*Threat expectancies*. Differences in pre-existing threat and reward expectancies of irritable youth compared to non-irritable youth may also contribute to activation of negative and positive valence and cognitive control systems, such as via early vulnerability factors and activation via setting factors. For example, some evidence points to irritable youth being more likely to inaccurately perceive the intensity of emotional stimuli, suggesting that pre-existing threat expectancies influence the performance of irritable youth (i.e., Levy et al., 2022; Stoddard et al., 2016)**,** and may also influence a child’s mood state. Indeed, prior studies have suggested that highly irritable youth may automatically identify stimuli as more threatening or hostile than non-irritable youth, reflecting a hostile attribution bias (Crick & Dodge, 1994; Leibenluft & Stoddard, 2013). Although some studies have also suggested that irritable youth demonstrate more inaccuracies in face emotion labelling in addition to requiring a greater degree of facial emotional intensity before they can accurately identify the emotion when compared to healthy controls, research in this area is sparse and has relied on irritability in the context of Severe Mood Dysregulation (SMD; characterised by severe irritability and low mood) (Guyer et al., 2007; Rich et al., 2008). Some irritable youth have been found to view neutral faces as more hostile or threatening (Crick & Dodge, 1994; Leibenluft & Stoddard, 2013; Stoddard et al., 2016), with hostile attribution biases suggested to arise from early vulnerabilities, such as parent-related factors (Griffith et al., 2021; Lee et al., 2017). In general, youth appear to preferentially process unexpected information, consistent with studies suggesting a general expectancy bias in individuals (Proulx et al., 2017). Thus, taken together, these findings reflect a vulnerability for irritable youth to preferentially attend to threat information as a result of the activation of expectancy-violating information, such as through the attribution of hostile intent. When such threat expectancies are activated (such as in specific setting conditions), the activation of negative valence systems is likely to occur.

*Reward expectancies.* Similar to threat expectancies, differences in pre-existing reward expectancies of irritable youth may also contribute to activation of positive and negative valence systems. Although initial errors in reward (or punishment) predictions may arise from early vulnerabilities as previously described (i.e., inconsistent parenting strategies may lead to children first developing erroneous expectancies about conditions under which they may receive a reward), youth prone to irritability also demonstrate deficits in their reinforcement learning over time i.e., probabilistic and reinforcement learning; reward prediction error (Brotman et al., 2017a, 2017b; Brotman et al., 2017a, 2017b; Meyers et al., 2017). For example, Dickstein et al. (2007) examined responses on a task involving youth aged 7 to 18 years learning to adjust to changes in previously reward responses, finding that youth with BD and SMD made more errors compared to healthy controls when tasks involved reversal learning. Thus, a child’s erroneous reward expectancies are likely to lead to situations that ‘set the scene’ for the activation of negative and positive valence and cognitive control systems in response to reward stimuli (Perlman et al., 2015; Rich et al., 2011), such as pre-existing beliefs about whether or not they will receive a reward and the development of habitual responding to previously rewarded situations. Taken together, early vulnerability factors (e.g., parenting-related factors) combined with setting conditions under which a child may expect to receive or not receive a reward may lead to overactivation or underactivation of the positive valence system, or overactivation of the negative valence system. These conditions of reward expectancies may be further compounded by setting factors, such as a child’s mood state, priming irritable youth to have increased difficulty making judgements in conditions of possible reward or threat, thus also evoking activation of positive and negative valence systems.

## Underlying Processes (C & D)

Numerous models have been proposed to explain overlapping aspects of self-regulation of emotion, cognition, and behaviour and as a result, a more unified approach to terminology has been recommended (see Nigg, 2017 for review). Therefore, we aim to examine the underlying cognitive control processes of irritable youth, rather than complex aspects of self-regulation or control of emotions and behaviour more broadly, such as those proposed in models of emotion regulation (i.e., Ochsner et al., 2012) and the RDoC domains of arousal/regulatory systems (NIMH, 2021).

Arising from the development of top-down executive functions (i.e., working memory, response selection; Nigg, 2017), cognitive control is thought to develop across childhood by the age of 10 years (Munakata et al., 2012) and allows for active maintenance of cognitive, behavioural, and emotional goals (Miller & Cohen, 2001; Nigg, 2017). Although in the present review, the focus is on conceptualising irritability specifically within middle childhood, given nascent research in this area, we also draw upon studies specifically examining irritability and DMDD from preschool to adolescence.

Recent conceptualisations of the processes leading to irritability implicate underling system activations via evidence of both threat and reward biases (Brotman et al., 2017a, 2017b). In the model proposed by Kircanski et al. (2019), reduced inhibitory control (as a facet of cognitive control) was suggested as an underlying cognitive process associated with greater expression of frustration and behavioural outbursts, thus implicating a broader self-regulation difficulty impacting goal maintenance. Further, Kryza-Lacombe et al. (2022) described the mitigating role of executive functioning (cognitive flexibility and inhibitory control) on emotion regulation in reward biases among irritable youth. Recently, Brænden et al. (2022) highlighted key themes as related to the RDoC domains that are implicated in irritability, including underlying negative valence systems (i.e., frustrative nonreward), positive valence systems (i.e., reward anticipation and responses), social systems deficits (i.e., interpretation differences, biases toward threat faces) and cognitive systems (i.e., cognitive control, inhibition). However, highlighted within Brænden et al’s (2022) review was equivocal results given the intersection of cognitive systems with many of the RDoC domains (i.e., positive and negative valence systems, systems for social processes) thought to underlie irritability.

Thus, the proposed model extends upon the earlier models and reviews and aims to conceptualise cognitive control in terms of proactive and reactive processes that are proposed to be influenced by positive and negative valence system activation and underlie the processing of threat and reward stimuli (Miller & Cohen, 2001; Nigg, 2017) (Fig. 1). That is, common themes across the literature on both threat and reward biases in irritable youth suggest differences in initial responding, allocation of attention, sustained attention, interferences in attention, and response inhibition, arising from inflexible employment of specific top-down and bottom-up cognitive processes. Thus, in explicating the underlying cognitive control processes, we apply Braver's (2012) Dual Mechanisms of Control (DMC) framework in the context of the RDoC domains of negative (i.e., threat biases) and positive (i.e., reward biases) valence systems and cognitive control processes, as characterised within Fig. 1.

### Dual Mechanisms of Control

As outlined above, the DMC framework draws together two distinct but interacting cognitive modes, including *proactive control* and *reactive control.* Consisting of goal-directed processing, proactive control involves top-down processing whereby individuals actively select goal relevant information in order to maintain their attention on task goals (i.e., anticipating and preventing possible interferences in attention prior to distraction occurring) (Braver, 2012; Braver et al., 2009). For example, within the classroom setting, engaging in proactive control may involve a student anticipating distraction from classmates and actively selecting to maintain focus on goal-relevant information (and thus they are more easily able to filter out expected distractions).

On the other hand, reactive control is the process of correcting a response following interference in a goal-directed activity (i.e., detecting and correcting interferences in attention once they have occurred), such as correcting and redirecting attention away from classmates following distraction. Successful task engagement is thought to arise from flexibly implementing both proactive and reactive control, with changes in task demands likely to influence the control process employed (Braver, 2012; Braver et al., 2009). Although greater resources (e.g., increased working memory load) are required to engage proactive control, the shift from youth spontaneously preferencing proactive control rather than relying on reactive control increases throughout childhood. However, once instructed to do so, children as young as preschool age are able to engage in proactive control (Gonthier & Blaye, 2022). For instance, anticipating possible interferences in attention from classmates (proactive control) may reduce the frequency of distraction, however reliance on redirection of attention back to a task following distraction (reactive control) is less cognitively demanding. Thus, overreliance or preferencing of one control process can lead to inflexible and inefficient employment of cognitive control, interfering in task outcomes.

Moreover, expectancy (arising from a combination of vulnerability and setting factors) is also thought to shift the control mechanism employed, with higher expectancy of potential interferences in tasks influencing the recruitment of proactive control (Braver, 2012; Burgess & Braver, 2010), particularly when sufficient benefit is thought to occur (i.e., rewards, avoidance of punishment) in return for the higher cognitive load required for proactive control (Grimshaw et al., 2018). As such, if a child *expects* to become distracted by students within the classroom setting and is highly motivated to maintain their attention on a task goal, they may be more inclined to employ proactive control at the expense of it requiring higher cognitive load. On the other hand, if a child is not expecting to become distracted by classmates and/or is not motivated to maintain attention on task goals, they may be more likely to rely on reactive control. Thus, flexibly shifting between the complementary modes of proactive and reactive control as required would be considered most optimal.

Individual differences are also thought to impact engagement of cognitive control processes, influencing preferred recruitment of proactive or reactive control during active task processing. Such individual differences include affect, threat sensitivity (Savine et al., 2010) and reward sensitivity (Jimura et al., 2010). For instance, in situations in which positive valence systems are activated and children are sufficiently motivated, it is likely that youth will preference proactive control. Indeed, overactivation of the positive valence system may also lead to an overreliance on proactive control and lead to inflexible recruitment (and thus challenges flexibly shifting to corrections via reactive control as needed). On the other hand, in cases of reduced motivation and/or high task demands, youth may preference reactive control, at the expense of task performance. Indeed, changes in setting factors (e.g., increasing task demands, changing contingencies) may also lead to activation of positive and negative valence systems, and thus impact flexible engagement in proactive and reactive control, as outlined below. Thus, as illustrated in Fig. 1D, differential employment of cognitive control processes of proactive and reactive control may explain current findings in threat and reward biases in irritable youth. In turn, these differences in processing appear to drive variations in the expression and progression of irritability in children (as seen in Fig. 1E). Given current research examining irritability has largely been driven by separating biases in threat and reward (Brotman et al., 2017a, 2017b; Kircanski et al., 2019), we thus draw upon these key bodies of research in the application of the DMC framework below as a theoretical avenue to assist conceptualisations of irritability in future research.

### Aberrant Threat Processing

Current research on irritability has utilised a range of tasks to assess threat biases in youth. Biases in processing threat information has further been examined with regard to neural processing changes in youth with heightened irritability, providing an index of how brain functioning under conditions of threat may impact behaviour and cognitive processes. Although a number of studies have examined threat biases within irritable youth, these studies have varied widely in their approach to assessment. Specifically, studies include both community and clinical samples of irritability, they have recruited on the basis of diagnoses which have irritability as a key symptom (i.e., DMDD, SMD), or as an associated feature (ADHD, anxiety), and have ranged in severity and age (i.e., infancy, middle childhood, adolescence). Although a considerable number of studies have examined threat biases in youth with psychopathology with irritability as a symptom or associated feature (e.g., ADHD, ODD), this review focuses on studies with direct measures of irritability, or disorders with irritability as a key component (i.e., DMDD, SMD). Studies of threat biases among irritable youth compared to non-irritable youth suggest differences in the interpretation of threat, allocation of attention toward threat, interference of threat information, and sustained attention on threat information. Within the context of the RDoC negative valence system, we draw upon the domains of acute threat (i.e., indexed via allocation of attention toward rewards, and interference of threat information) and sustained threat (i.e., indexed via interference of threat information and sustained attention). Although frustrative nonreward (i.e., indexed via punishment salience and interference, withholding of rewards salience and interference) in the context of the RDoC domains (and Fig. 1C) is placed within the negative valence system, this is discussed below as a separate construct due to its overlap with reward processing. Despite current evidence highlighting differences within threat biases for irritable youth, we emphasise a common theme of inflexible or inefficient employment of proactive and reactive control as a key underlying cognitive process reflected in threat biases.

*Attention allocation.* Across prior research, threat attention biases have been found to be a feature of irritable youth (Deveney et al., 2020; Hommer et al., 2014; Salum et al., 2017). Specifically, alterations in neural activation have been found during emotion labelling tasks of ambiguous faces in 71 youth aged 9- to 21-years-old with DMDD when compared to healthy controls and those with bipolar disorder (BD) (Wiggins et al., 2016), suggesting overactivation of acute treat in the negative valence system. Within a community sample of irritable youth, Salum et al. (2017) assessed the visual allocation of attention to threat stimuli in a sample of in a sample of 6- to 12-years-old using the visual dot-probe paradigm. Children who were often irritable had greater attentional biases towards threat relative to neutral faces when compared to non-irritable children. Hommer et al. (2014) found similar results in a clinical sample of youths aged between 7- to 17-year-olds with SMD with results indicating that the more SMD and depressive symptoms that youth had, the greater their attentional bias toward threat. Notably, these results were also found after controlling for comorbidity and medication use, demonstrating that irritability plays a role in attentional biases, despite its high co-occurrence with other disorders. Together, the faster reaction times to probes when congruent with threat information combined with slower corrections to threat-incongruent information observed in irritable youth relative to healthy youth in these studies (Hommer et al., 2014; Salum et al., 2017) suggests an overactivation of the negative valence system (i.e., acute threat; Fig. 1C). This activation is thought to lead to irritable youth preferencing reactive control and redirecting attention following distraction, rather than proactively selecting to allocate attention to goal-relevant information (i.e., proactive control) thus leading to greater susceptibility to distraction by threat information.

Conversely, Deveney et al. (2020) investigated visual attention allocation using a dot-probe task with emotional faces whilst measuring event-related brain potential indices of attention. Children’s correct responses earned them gold coins which were exchanged for a prize at the end of the task. Here, findings from Deveney et al. (2020) suggest that when irritable youth can earn rewards based on their correct responses, these biases toward threat faces are not observed (i.e., no differences in reaction time and accuracy). Instead, it is likely that under conditions of reward which increase motivation, irritable youth can preference more cognitively demanding methods of cognitive control (i.e., proactive control) to maintain their attention on task demands and flexibly shift to correct any interferences in attention once they occur (i.e., reactive control), thus in turn improving task performance. However, in doing so, irritable youth demonstrate greater neural responsivity, reflecting higher cognitive load to engage these processes. This pattern highlights the interplay of both positive and negative valence system activation, in that youth demonstrate a preference for proactive control due to increased motivation (i.e., positive valence system activation) which may override or reduce the impacts of the negative valence system activation induced via threat information and allow more flexible recruitment of both proactive and reactive control. Given little research conducted in this area, further studies are required to understand under which conditions positive valence system activation may override or negate the effects of negative valence systems, and vice versa.

*Threat interference.* Maladaptive employment of cognitive control is further seen in the capacity of irritable youth to ‘filter out’ distracting information. This was demonstrated in Stoddard et al. (2017), in which youth with anxiety, DMDD, and/or ADHD, and healthy controls were required to label the gender of faces presented at different emotional intensities. Findings suggested that more severe irritability was associated with lower accuracy in labelling the gender of intensely angry faces, indicating that these youth have difficulty inhibiting threat-related information in order to maintain task goals. Further, heightened irritability and/or anxiety was associated with differential neural activation when presented with angry and happy faces compared to fearful faces. Thus, these findings suggest maladaptive employment of cognitive control as seen in Fig. 1D, in that youth with more severe irritability have greater reliance on correcting attention once distracted (reactive control) as opposed to attempting to actively maintain focus on filtering out distracting information (proactive control), also reflecting overactivation of the negative valence system.

*Sustained attention on threat.* Biases in sustained attention on threat information is also observed, indicating that irritable youth may be at greater risk of recognising and then maintaining their attention on threats within their environment, and thus limiting their capacity for cognitive control when presented with a goal-directed task. Illustratively, Harrewijn et al. (2021) found that youth with anxiety and irritability in the context of ADHD, DMDD, and/or ODD looked at negative compared to non-negative faces for longer durations in the happy-angry condition compared to the happy-sad condition on an eye-tracking task. Higher negative affect (a latent factor composed of irritability and anxiety) was associated with longer durations of looking at negative compared to non-negative faces. Moreover, Kircanski et al. (2018) examined attention to threat using fMRI on a dot-probe task in 8- to 18-year-olds with varying levels of irritability in a transdiagnostic sample. Higher parent-reported irritability was associated with greater neural engagement to maintain control during threat-incongruent trials when requiring attending away from threat. Similarly, Kryza-Lacombe et al. (2020) found greater neural activation and more pronounced fluctuations in neural activation in irritable versus non-irritable youth when required to shift attention away from threat stimuli on a dot-probe task, despite no differences in overall accuracy and speed. Thus, findings of Kircanski et al. (2018) and Kryza-Lacombe et al. (2020) suggest that although task performance may be similar (i.e., reaction time), greater neural engagement is needed to employ proactive control in irritable youth when required to allocate attention away from threat information and maintain attention on task goals. Together, these findings suggest that more severe irritability is associated with sustained attention on negative emotional stimuli relative to non-negative faces, suggesting greater difficulties disengaging attention from salient information, and/or greater attentional capture from emotionally salient information. Thus, findings point to irritable youth being more prone to selecting information and sustaining attention on emotional (compared to non-emotional) faces. In turn, this arguably increases their susceptibility to become frustrated by contextual factors (i.e., negative valence system overactivation) and thus reducing their capacity to recruit proactive control when required to engage in a specific task goal.

*Summary.* Drawing these findings together, it is clear that there are abnormalities in processing threat stimuli in irritable youth. Findings point to patterns of increased attention toward threats, greater difficulty ignoring threat distractors, and problems redirecting attention back to task goals when distracted by threats. Thus, aberrant threat processing with regard to responding to acute threat and sustained threat (i.e., RDoC domains; Insel et al., 2010) appears to play a key role in irritable youth, but findings point to potential differences in the cognitive processes underlying these biases (i.e., Fig. 1D). Specifically, youth with high levels of irritability are more prone to emotional interference, both allocating and maintaining attention towards threat information, and as a result leading to overactivation of the negative valence system. Given that individuals with high irritability also require greater neural resources to employ more cognitively demanding modes of cognitive control (i.e., engage in flexible recruitment of proactive control), they may preference less cognitively demanding methods of control (i.e., reactive control), particularly when the outcomes of engaging in this higher cognitive load needed to flexibly shift between both proactive and reactive control is not valuated to be sufficiently rewarding (demonstrating an underactivation of the positive valence system). On the other hand, youth may recruit these more cognitively demanding processes under conditions of possible reward through positive valence system activation.

### Frustrative Nonreward

At the intersection of both threat and reward processing, some findings also point to differences in the ability of irritable youth to maintain attention on task goals due to the influence of reward omission and removal of rewards (Fig. 1C). Utilising the Posner task, Rich et al. (2011) found that youth with SMD were more unhappy and agitated than controls in response to negative feedback. SMD youth had greater neural activation in the anterior cingulate cortex and medial frontal gyrus following negative feedback, suggesting that youth with SMD are more sensitive to negative feedback. Similarly, Rich et al. (2007) also compared performances on a Posner task in which children with SMD and BD both self-reported increased frustration following the rigged punishment trials (i.e., loss of previously rewarded money). Although both clinical groups had significantly faster reaction times on post-punishment trials compared to controls in both frustration and non-frustration conditions, youth with SMD were significantly less accurate in indicating the location of the target across all trials in comparison to both BD and control groups. Changes in Event Related Potential (ERP) amplitude of N1 suggested that children with SMD have deficits in the initial attention allocation across frustration and non-frustration trials, likely interfering in their accuracy across trials, suggesting initial overactivation of the positive valence system. However, given youth with greater irritability had reduced accuracy on no-go trials (relative to the go trials), faster go reaction times (relative to no-go reaction times), and larger amplitudes during the frustration conditions compared to the non-frustration conditions, findings suggest that youth with heightened irritability employ greater neural resources to recruit proactive control and thus respond more quickly when rewards are blocked. However, such employment of proactive control is inflexible, leading to deficits in their ability to monitor unexpected information to respond appropriately (i.e., inhibit required responses) to task demands. Thus, youth with high irritability were not only more frustrated when experiencing punishment but may also be impacted with respect to effective methods of task engagement (i.e., speeding up; Rich et al., 2007). This pattern of results suggests that youth with heightened irritability engage in inflexible methods of cognitive control resulting from initial overactivation of the positive valence system (i.e., increased responsiveness to reward, reward overvaluation), and thus sustained employment of proactive control likely enhances their speed following frustrating feedback, and as a result impacts overall task accuracy. In turn, this may lead to negative valence system overactivation via frustrative nonreward. Thus, prior research suggests an interplay in the processing of threat and reward stimuli in which frustrative nonreward is activated, leading to further impacts on the effective and flexible recruitment of proactive and reactive control among irritable youth.

### Aberrant Reward Processing

Similar to research in threat processing, various tasks have been used to provide an indication of aberrant reward biases in irritable children. Many studies have utilised variations of the Posner task to index responses to reward, punishment, and reward omission through behavioural and/or neural responding. Further, studies such as the reversal task and the go/no-go task provide indications of the ability of irritable youth to inhibit responding in the context of reward, reward omission, and punishment. Samples of reward processing in irritability have largely focused on clinical populations of youth in middle childhood, or irritability in very young children (i.e., under 6 years of age). Research into reward processing suggests differences in the salience of reward and punishment, attention toward reward-related cues, reward interference, and response inhibition in irritable youth compared to non-irritable youth. Within the context of the RDoC positive valence system, we draw upon the domains of reward undervaluation (i.e., indexed via reward salience), reward responsiveness (i.e., indexed via attention toward reward/reward omission), reward overvaluation (i.e., indexed via attention toward reward/reward omission, reward interference), and reward learning (i.e., indexed via response inhibition). However, similar to threat processing research, differential activation of these domains influence employment of proactive and reactive control.

*Reward salience (undervaluation).* Although prior research has focused on threat and reward processing in irritability, few studies have examined attention and task engagement under condition of low or now reward (i.e., positive valence system underactivation). In two studies that have examined attention towards positive stimuli, neither Hommer et al. (2014) nor Salum et al. (2017) found biases in attention allocation to happy relative to neutral faces. The absence of attention biases toward happy relative to neutral faces may indicate that happy faces are not considered salient rewards for irritable youth or may be suggestive of reward undervaluation. Dickstein et al. (2007) also found that SMD and BD youth were less accurate and slower relative to a control group on change trials during a Change Task. Similarly, Cardinale et al. (2023) found that during a Flanker Task administered at 15 years old with youth with developmental trajectories of irritability and anxiety, irritability was associated with greater neural responding to conflict (i.e., responses on incongruent versus congruent trials) and longer reaction time differences between congruent and incongruent responses, whereas anxiety was associated with decreased neural responding. Thus, findings suggest that youth with more severe irritability may preference reactive control (therefore reducing their speed and accuracy) when required to engage in tasks that are more cognitively demanding, particularly without the potential to earn a reward (i.e., positive valence system underactivation; Fig. 1C). However, the extent to which task performance may be intrinsically rewarding for youth requires much further research. For instance, Fishburn et al. (2019) examined a group of preschool-aged children in a non-clinical sample on go/no-go task, finding that the temperamental domain of anger/frustration was associated with activation related to slowing down on ‘no go’ trials compared to speed on ‘go’ trials. Specifically, as irritability increased in these youth, activation in the PFC also increased during inhibitory control and thus although community populations of youth with a disposition to experience irritability may require greater cognitive resources to employ cognitive control for the purposes of maintaining attention on a task goal under, these youth are able to recruit proactive control to maintain attention on task goals (albeit reducing their speed). However, it was noted that in this study, youth were engaged via delivery of a story as part of the task goals, which may arguably capture their attention and engagement, and thus be intrinsically rewarding for children. Indeed, one study found that in a group of 208 treatment-seeking 6- to 12-year-olds with DMDD, ADHD, ODD, or DMDD and ADHD, executive functioning (i.e., cognitive flexibility, emotion control) was found to differ in testing situations compared to parent reporting for children with DMDD. Such differences were posited to arise in emotion-neutral situations in which children with DMDD have the capacity to problem solve and reason more flexibly. However, in emotionally demanding situations, children with DMDD may not have the regulatory capacity to cope (Brænden et al., 2023). Thus, although further research is required to characterise the unique relationships between irritability and neuropsychological functioning, these findings highlight potential differences in setting factors (i.e., task demands) and evaluation of tasks as intrinsically rewarding, and thus influencing preferential employment of cognitive control processes, such as increased ability of irritable youth to flexibly recruit proactive control where tasks may be less cognitively demanding. Thus, these findings suggest differences in the valuation of rewards for irritable youth, highlighting that some tasks may be more intrinsically rewarding for children and motivating youth to employ more proactive methods of cognitive control. However, for some youth prone to irritability, they rely on reactive control, suggesting an undervaluation of either intrinsic or extrinsic rewards and thus underactivation of their positive valence system.

*Reward salience (overvaluation).* Although the salience of rewards may improve the motivation of highly irritable youth to engage more efficient cognitive control processing, this can become inflexible or limited in the presence of reward overvaluation and/or punishment or frustration for these youth. Kessel et al., (2016a, 2016b) found that of 425 participants, children with severe DMDD symptoms at age three were associated with increased sensitivity to rewards, indexed by their greater EEG positivity response during a reward task at age nine. Despite no differences in overall performance for those with more severe DMDD symptoms when compared to those with less severe symptoms, and no differences in neural responding in response to punishment, greater neural responsiveness to reward suggests that individuals with severe DMDD respond more positively to the presence of rewards. Moreover, Perlman et al. (2015) supported these findings in a sample of children with severe irritability when compared to 28 healthy children on a frustration task. Findings indicated that the group with more severe irritability displayed differential neural activation during both losing trials (i.e., blocked goals) and reward trials compared to healthy children, although no differences in reaction times were found. Taken together, these studies suggest that children with more severe irritability are more sensitive to rewards, as indexed by greater EEG positivity, suggesting positive valence system overactivation (i.e., reward overvaluation, reward responsiveness), and experience greater frustration when rewards are omitted or removed (i.e., frustrative nonreward). In turn, this elevated responsivity to rewards may influence irritable youth to engage in proactive methods of cognitive control to meet task demands. However, this may hinder performance when presented with frustrating stimuli or settings, such as reward omission, in which frustrative nonreward is activated and thus proactive methods of cognitive control become inflexible or too cognitively demanding to allow flexible shifting between proactive and reactive control (presented in Fig. 1D).

*Attention toward reward.* Similar to findings within threat processing, youth with heightened irritability sustain their attention on rewards, thus limiting their capacity to employ proactive control. In a younger sample, He et al. (2013) assessed 311 Chinese school children in three separate samples completing mood induction prior to the affective Posner task. Similar to in the study conducted by Rich et al. (2007), He et al. (2013) found that children acutely experiencing anger and children prone to anger allocated their attention toward reward compared to punishment cues more quickly (i.e., overactivation of the positive valence system; reward responsiveness, reward overvaluation). However, when frustrated, irritable youth have difficulty adjusting their performance to changes in task demands and have less capacity to employ proactive control (i.e., reflecting frustrative nonreward). In addition to these findings, children experiencing anger following the emotion-induction task also had difficulty redirecting their attention back to the task after orienting their attention towards reward cues, particularly after negative feedback. Thus, these findings suggest that highly irritable youth differentially allocate their attention in the context of reward and punishment, likely suggesting greater reward responsiveness and overvaluation of rewards, which in turn may lead to inflexible recruitment of proactive control. However, once frustrated, these youth demonstrate impacts on their ability to sustain their attention on task goals (i.e., employ proactive control) compared to non-irritable youth, reflecting frustrative nonreward. This further highlights the link between the setting conditions (Fig. 1B) and employment of cognitive control processes (Fig. 1D), whereby rewards (or the omission of rewards) within a child’s environment due to changes in task demands influences recruitment of proactive and reactive control.

*Reward interference.* Irritable youth also appear to have deficits in their ability to redirect attention back to task goals due to reward omission or punishment, suggesting difficulties in flexibly employing both proactive and reactive control as required. For example, Deveney et al. (2013) examined responses of 19 children with SMD and 23 healthy controls aged 8- to 17-years-old on an affective Posner task. Irritable youth reported higher levels of frustration during the frustration task (i.e., frustrative nonreward; Fig. 1C). Initially, no differences in speed or accuracy were observed between groups, however during the frustration task, participants with SMD were slower than healthy controls on incongruent trials, indicating likely overreliance on proactive control (i.e., not correcting interferences in attention by employing reactive control) and greater interference from frustration. Moreover, Kryza-Lacombe et al. (2021) found higher irritability was associated with altered processing during reward anticipation and reward omission in a sample of 52 9- to 19-year-olds. Results of these studies both point to youth with higher irritability having difficulty disengaging their attention from cues of punishment or blocked rewards (i.e., difficulty employing reactive control), thus interfering with task goals. These results were similar to those by Tseng et al. (2018) in that although no differences in performance (reaction time and accuracy) were found, higher irritability was associated with increased activation across a number of neural regions following rigged compared to positive or no feedback, particularly for younger youth (8–11.5 years old). These findings suggest that younger youth with increased irritability require greater neural resources to maintain task goals and shift their attention back to task demands once frustrated (i.e., employ reactive control). Together, findings suggest that although irritable youth may engage in proactive methods of control when motivated by potential rewards due to overactivation of their positive valence system (i.e., increased reward responsiveness, reward overvaluation), this requires greater neural resources and/or it may be rigidly employed. Further, once frustrated, these youth also appear to have difficulty flexibly correcting interferences in attention (i.e., employing reactive control), impacting on their task performance.

*Response inhibition.* With regard to the responses to reward stimuli, studies also suggest that youth with heightened irritability have difficulty inhibiting both cognitive and behavioural responses when attempting to achieve a desired goal. These differences in response inhibition highlight reduced employment of reactive control due to an overreliance on proactive control. First, Adleman et al. (2011) examined responses on a simple reversal task of 82 youth with SMD, BD and healthy controls. Youth with SMD made more errors across all trials than both BD and healthy control groups. Moreover, youth with SMD had decreased neural responsiveness in the inferior frontal gyrus, indexing the ability to maintain attention and inhibit both cognitive and behavioural responses when attempting to achieve a desired goal (e.g., receive the reward). Therefore, the findings suggest that youth with SMD have difficulties correcting interferences in attention from negative feedback, further demonstrating an over-reliance on proactive control and inefficient employment of reactive control. In a younger sample, response inhibition was assessed in a sample of 46 children aged 4- to 7-years-old. Children completed a go/no-go task with EEG data and youth with greater irritability had reduced accuracy on no-go trials (relative to the go trials) and faster go reaction times (relative to no-go reaction times) during the frustration conditions, compared to the non-frustration conditions. Higher irritability was also associated with larger amplitudes during frustration. Third, response inhibition was also assessed by Seymour et al. (2020) in a sample of 105 youth aged 8- to 12-years-old with ADHD and typically developing controls, ranging in irritability severity. On a reward-based frustration go/no-go paradigm, frustration levels reduced response inhibition (i.e., participants had higher commission errors, variability in responding, and attentional lapses) across all conditions. However, given that youth with higher irritability reported higher frustration during the task, the findings suggest that these youth may be more susceptible to frustration when a reward is blocked, leading to reduced response inhibition. Together, these findings suggest that when motivated by a reward (i.e., overactivation of positive valence system via reward overvaluation), youth with greater irritability are more inclined to inflexibly recruit proactive methods of cognitive control, hindering their ability to respond appropriately to detect and correct interferences in their attention (i.e., reactive control).

*Summary.* Taken together, this nascent but growing body of research suggests that irritable youth not only exhibit threat biases, but they also demonstrate differences in the processing of rewards as well. Highly irritable youth demonstrate greater sensitivity to the possibility of rewards, are more frustrated when experiencing punishment or blocked rewards, and differ in their expectations of rewards and punishment, as suggested in prior research (Brotman et al., 2017a, 2017b; Kircanski et al., 2019), reflecting positive valence system overactivation (i.e., reward overvaluation, reward responsiveness), differences in reward learning, and frustrative nonreward (Fig. 1C). Such sensitivity to rewards likely influences irritable youth to engage in proactive methods of cognitive control and improve performance. However, in instances where youth become highly frustrated, such as in response to increased demands of setting factors (e.g., increasing task demands; Adleman et al., 2011; Deveney et al., 2013; Rich et al., 2007) or a negative mood inducing event prior to the task (i.e., He et al., 2013), reduced capacity to employ proactive control (and thus reduced performance) is observed due to overactivation of the negative valence system. Alternatively, proactive employment of control can become rigid, with youth unable to flexibly employ both proactive and reactive control as required, leading to reduced redirection back to task goals (i.e., reactive control, in Fig. 1D). On the other hand, youth who experience high irritability also demonstrate undervaluation of rewards (Dickstein et al., 2007), leading to underactivation of the positive valence system. Such underactivation appears to be linked to youth then preferencing less cognitively demanding control systems (reactive control). Together, these findings indicate that although potential rewards can enhance motivation to recruit more cognitively demanding control processes (i.e., proactive control) and flexibly shift between proactive and reactive control, once reward contingencies change, or when the demands of setting factors increase, these approaches can become inflexible or less effective in response to the specific task requirements.

## Expression and Progression (E)

*Tonic and phasic irritability. *As noted, recent conceptualisations of irritability have suggested interrelated yet distinguishable expressions of irritability, namely tonic and phasic irritability (Copeland et al., 2015). Tonic irritability (Fig. 1, pink border) has been described as persistently grouchy or grumpy mood, with youth being touchy or easily annoyed. Conversely, phasic irritability (Fig. 1, blue border) is defined as developmentally inappropriate outbursts or tantrums (Copeland et al., 2015; Vidal-Ribas & Stringaris, 2021). Although tonic and phasic irritability have been identified as unique and discrete constructs, they are also interrelated in that phasic irritability has been linked to the development of tonic irritability, and tonic irritability has been linked to the development of phasic irritability (Copeland et al., 2015; Moore et al., 2019). Indeed, both tonic and phasic expressions of irritability have been associated with disruptions in functioning and poorer outcomes for youth (Copeland et al., 2015), associated with different patterns of psychopathology (as seen in Fig. 1E).

*Outcomes*. Depicted in Fig. 1C are the positive and negative valence systems, as related to the underlying cognitive processes of proactive and reactive control, and tonic and phasic irritability that are both observed as core components in DMDD. Although DMDD has been more closely associated with phasic presentations of irritability (Moore et al., 2019), youth who have three or more developmentally inappropriate temper outbursts per week (i.e., phasic irritability) in addition to persistent irritable mood between outbursts (i.e., tonic irritability) satisfy diagnostic criteria for DMDD (American Psychological Association, 2000). Given this interconnected nature of tonic and phasic irritability, DMDD at the age of 6 years old predicted significantly lower global functioning and increased comorbid psychopathology, including increased symptoms of ADHD, disruptive behaviour disorders, and depression (Dougherty et al., 2016). Further, persistently irritable youth (both tonic and phasic irritability) demonstrate greater risk for depression at 13 years old (Orri et al., 2019) and higher suicidality risk in adolescence (Forte et al., 2021; Orri et al., 2019).

Despite irritability being a core feature of DMDD, not all children with high irritability satisfy diagnostic criteria for DMDD. Certainly, both tonic and phasic irritability have been observed in the context of ODD, which when compared to DMDD is considered less severe with respect to irritability symptoms. As illustrated in Fig. 1E, youth with ODD may present with either phasic *or* tonic irritability, rather than requiring the presence of both (American Psychological Association, 2000). In addition to also displaying phasic irritability, youth with internalising disorders (i.e., depression and anxiety) more commonly exhibit tonic irritability (as presented in pink in Fig. 1), as irritable mood is a common symptom of these disorders within the DSM-5 (American Psychological Association, 2000; DeGroot et al., 2023; Stringaris, 2011). Indeed, within an adolescent sample, youth with higher tonic irritability were observed to develop later depressive disorders and anxiety (Silver et al., 2021). However, some recent evidence has suggested greater impacts on functioning for youth with selective mutism who display phasic forms of irritability (Freitag et al., 2023), indicating the need for further studies parsing these constructs in the context of youth psychopathology. On the other hand, youth with ADHD demonstrate greater phasic irritability (i.e., increased outbursts and aggression) rather than persistently irritable mood (Cardinale et al., 2021; DeGroot et al., 2023). Moreover, youth with more severe phasic expressions of irritability demonstrated associations with the development of externalising disorders (Silver et al., 2021), as presented in blue in Fig. 1. Therefore, alongside varying concurrent patterns of psychopathology with tonic and phasic irritability, youth also demonstrate associations with psychopathology and poorer outcomes in the longer-term. Although tonic and phasic irritability are depicted in Fig. 1 as associated with patterns of psychopathology and underlying cognitive processes, current research suggests that these constructs are not mutually exclusive and that instead they commonly co-occur (Copeland et al., 2015; Moore et al., 2019). Indeed, although both tonic and phasic irritability are interrelated, and together they increase the risk of poorer youth outcomes, separately these expressions of irritability may also be associated with distinct outcomes (Leibenluft & Kircanski, 2021). Further work is still required to understand the unique longitudinal outcomes associated with both tonic and phasic irritability in childhood.

## Conclusions

Although the mechanisms of threat and reward biases have been previously been included in pathophysiological models of irritability in youth (Brænden et al., 2022; Brotman et al., 2017a, 2017b; Kircanski et al., 2019), the differential pathways of positive and negative valence system activations that may drive maladaptive activation of cognitive control (i.e., proactive and reactive control) processing differences among irritable youth is not clearly understood or explained. As such, we present a novel and expanded framework of irritability in which we have drawn together a summary of evidence regarding vulnerability factors for irritability (Fig. 1A) and how this likely interacts with setting conditions (Fig. 1B), thus leading to differential positive and negative system activation (Fig. 1C) and accentuating differential recruitment of cognitive control processes (Fig. 1D) and contributing to the expression and differential progression of irritability (Fig. 1E).

Findings point to clear processing differences for irritable compared to non-irritable youth under threat and reward conditions. However, these conditions also appear to interrelate, with potential rewards increasing an individual’s motivation to recruit more cognitively demanding methods of control (i.e., proactive control), such as via positive valence system activation or overactivation. On the other hand, the presence of threat and reward appears to reduce the capacity of irritable youth to employ proactive and reactive control effectively and flexibly (i.e., negative valence system overactivation; positive valence system underactivation). Indeed, although the availability of rewards may enhance the ability of irritable youth to engage in tasks, once contingencies change or rewards are removed, activation of the negative valence system (i.e., via frustrative nonreward) leads to more difficulty for youth to engage flexibly in both proactive and reactive control and thus is likely to lead to phasic outbursts. Moreover, highly irritable youth also demonstrate an overall requirement for increased neural resources in order to employ proactive control, likely leading to increased tonic irritability. These cognitive control differences therefore explain the differences in outcomes and expression observed among irritable youth compared to non-irritable youth. In particular, when interacting within a social context, irritable youth likely trigger hostile interactions with their peers (Brotman et al., 2017a, 2017b; Forte et al., 2021), likely arising from their sensitivity to threats and rewards and their reduced capacity for flexible cognitive control processing in the context of threats or rewards. Such differences evoking poor interactions with peers may involve a higher sensitivity to minor threats (negative valence system overactivation; e.g., name calling), greater competition with peers when a potential reward is involved (positive valence system overactivation; e.g., difficulties with turn taking or impulsivity), increased frustration when not receiving a desired reward (frustrative nonreward; e.g., phasic expressions of irritability when not winning a game), or reduced motivation when there are no potential rewards (e.g. positive valence system underactivation; reduced effort in challenging tasks) and longer-term tonic irritability. As such, these pathways of system activation impacting on effective and flexible recruitment of proactive and reactive control are likely to exacerbate early vulnerabilities (such as parent–child relationships and hostile attribution biases) and lead to ingrained and maladaptive responses to proximal setting factors (i.e., fatigue, mood state). Over time, this perpetuation of activation of underlying systems and processes is likely to lead to greater functional challenges for irritable youth and longer-term psychopathology. Illustratively, Sorcher et al. (2022) found that early irritability increases the risk for social difficulties in the longer-term. Indeed, Barclay et al. (2022) found that low social skills mediated the interaction between childhood irritability and adolescent internalising problems. In a sample of 739 8- to 11-year-olds, physical victimisation and peer rejection were found to exacerbate symptoms of ADHD and irritability, both concurrently and in the longer-term (Bellaert et al., 2023). Moreover, Barker and Salekin (2012) suggested that youth with high irritability and peer victimisation at age 10 was associated with increased callus-unemotional traits and internalising difficulties at age 13. Such interactions with peers over time combined with reduced capacities to self-regulate emotional and behavioural control, likely serve to increase risk for psychopathology in the long term (Orri et al., 2019).

Moreover, differences in underlying cognitive control processes may help to explain multifinality associated with early irritability symptoms. For example, Kessel et al. (2016) examined longitudinal associations of parent-reported irritability at age 3, with performance on a go/no-go task with continuous EEG monitoring at age 6. Higher irritability at age 3 and enhanced negative change in error-related negativity (ERN) at age 6 predicted more internalising symptoms at age 9. Conversely, blunted change in ERN at age 6 predicted more externalising symptoms among irritable youth. Such findings may arise due to underlying pathways of system activation (i.e., positive valence, negative valence, and cognitive systems) in which irritable youth may inflexibly engage cognitive control modes in an attempt to reduce potential interference from threats or inhibit phasic irritability, and as a result likely experience more tonic forms of irritability (pink border, Fig. 1). Intensive threat or behavioural monitoring over time is likely associated with increases in internalising symptoms in the longer term (i.e., depression). Alternatively, some youth who are prone to irritability may experience difficulties monitoring their performance and/or recognising the impact of their behaviours on others (perhaps resulting from difficulties recognising the emotions of others, particularly at low intensities) (Guyer et al., 2007; Rich et al., 2008), and may thus display more phasic irritability (i.e., temper tantrums or outbursts; blue border, Fig. 1) and externalising difficulties in the long term. These challenges with downregulation intersect with the wider RDoC domains of arousal/regulatory systems (i.e., arousal) and systems for social processes (i.e., perception and understanding of others) (Insel et al., 2010). Thus, differences in irritable youth with respect to their preferential recruitment of cognitive control processes during goal-directed tasks may be associated with variability in the expression of irritability and its associated psychopathology, and poorer functional outcomes and longer-term psychopathology.

## Implications, Limitations, and Future Directions

Building on prior proposed models and conceptualisations of irritability (Brænden et al., 2022; Brotman et al., 2017a, 2017b; Kircanski et al., 2019) this expanded theoretical framework highlights reward and threat processing as putative treatment targets. At the same time, it also recognises the implications of various setting factors, some of which may be intractable, and their role in stoking how irritable youth respond to environmental stimuli and demand triggers. This understanding in turn may lead to purposeful and specific investigations into irritability and how it contributes to psychopathology in youth across varied environments, elucidating targets for the treatment of irritability in children. Indeed, given gaps in our current understanding of aspects of childhood irritability (e.g., the role of protective personality characteristics, the role of expectancies in threat processing), this framework further highlights avenues for needed investigation. At the same time, this theoretical and conceptual review has limitations that warrant further discussion and need for future research. Although we have examined irritability in a variety of contexts, such as in clinical samples of DMDD, community samples, and in the context of co-occurring psychopathology (i.e., anxiety), many childhood disorders which have irritability as a symptom have not been included here. Such disorders (i.e., PTSD, childhood mania) require subsequent investigation regarding the applicability of this theoretical conceptualisation. Moreover, given that this is indeed a theoretical review, tasks designed to directly investigate underlying cognitive processes such as proactive and reactive control (e.g., the AX-CPT; Braver, 2012; Gonthier et al., 2016) are needed in order to provide empirical data to support or falsify the proposed framework. As a result, future research should consider tasks that directly map onto these underlying cognitive processes to better assess this theoretical framework and understand their recruitment in the context of various setting factors. Specifically, youth with heightened irritability demonstrate a general reliance on reactive control, leading to increased distraction from task goals, particularly under conditions of threat (i.e., overactivation of the negative valence system) or when setting factors are negatively impacting (e.g., task demands increase, increased frustration). When irritable youth are presented with rewards, these youth demonstrate more proactive control methods likely due to overactivation of the positive valence system, however this can be inflexible or require greater recruitment of neural resources to employ (and thus limit their capacity for continued recruitment of proactive control), particularly under conditions which activate frustrative nonreward. At the same time, it remains unclear the varying degrees to which setting factors and potential threats and rewards may interact to influence youth with heightened irritability—that is, how combinations of positive and negative setting factors together may influence cognitive control capacity, and how this may be expressed with regard to tonic and/or phasic irritability and its progression over the longer term. Consequently, further understanding of these interactions between setting conditions leading to activation of these systems, the recruitment of cognitive control, and associations with tonic and phasic presentations of irritability in both community and clinical samples, may prove advantageous for our understanding of interventions that may reduce irritability in youth. Additionally, further research into the longitudinal associations between these underlying cognitive processes and the development of psychopathology in youth is required to understand how these cognitive processes may map onto youth with less severe trajectories of irritability (Orri et al., 2019; Pagliaccio et al., 2018; Wiggins et al., 2014). Such future research may also consider wider application of underlying cognitive control processes to youth irritability, such as the systems for social processes (i.e., RDoC domains mediating interpersonal settings; Insel et al., 2010). Indeed, understanding these pathways in relation to underlying cognitive control processes will help highlight critical opportunities for future treatment research.

Advancing our understanding of the processes underlying irritability provides pathways for novel treatment research (i.e., targeting underlying attention biases; Brotman et al., 2017a, 2017b). As such, attention training may provide a fruitful avenue for the treatment of irritability in youth by modifying attention biases for threat (such as in the treatment of anxiety disorders; Waters et al., 2013, 2015). Further, exposure to frustration-inducing conditions may provide opportunities to modify reward contingencies (i.e., expectancies) and condition more effective methods of regulating emotional and behavioural outbursts (Kircanski et al., 2019). Indeed, recent studies (Grossman & Ehrenreich-May, 2020; Hawks et al., 2020) have found preliminary evidence for modular cognitive behavioural therapy (CBT) as a transdiagnostic intervention that may assist in the treatment of irritability. Naim et al. (2023) found further preliminary evidence for exposure-based CBT with parent-management training for severe irritability in children, with results suggesting stronger effects in targeting temper outbursts compared to irritable mood. Although these results highlight the utility of exposure-based approaches in the treatment of irritability, setting factors, differential activation of positive and negative valence systems, and inflexible and ineffective cognitive control recruitment are key factors that appear to interact to negatively influence poorer youth outcomes and expressions of irritability and thus further treatment avenues are also implicated. Specifically, in training youth to attend more flexibly to positive environmental cues (i.e., via positive search training), youth may be less susceptible to negative setting factors that may hinder their performance and thus improve more effective engagement of cognitive control whilst engaging in a goal-directed activity (i.e., reduced negative valence system overactivation). Further, some recent studies have highlighted mindfulness training as an effective approach to enhancing flexible cognitive control engagement. For example, in a sample of 94 undergraduate students, a mindfulness intervention group (compared to non-intervention and a passive relaxation group), demonstrated more flexible engagement in proactive and reactive control modes on the AX-CPT at post-intervention (Chang et al., 2018). Although these findings were within a sample of undergraduate students, mindfulness training shows some promising treatment indicators for irritable children. Illustratively, children with DMDD have demonstrated reduced irritability following Dialectical Behaviour Therapy which incorporates mindfulness training (Perepletchikova et al., 2017). Thus, findings point to interventions which include mindfulness components as one possible avenue for bolstering treatment for irritability in youth. Future research is required to fully investigate the efficacy of mindfulness training in order to characterise changes in flexible recruitment of proactive and reactive control in irritable children. Although prior research has highlighted the value in psychopharmacology for irritability, such as in ADHD (Coghill et al., 2014), direct targeting of cognitive control processes (i.e., recruitment of proactive and reactive control) in irritability through medication management requires further investigation. In particular, some studies (Posner et al., 2011; Schulz et al., 2018) have found psychostimulants to disrupt neural connections that may underlie emotional processing within adolescent and adult ADHD samples, thus calling for the need to examine the impact of these treatment approaches for irritable youth. Taken together, our framework highlights key opportunities for further investigation into the treatment of irritability in youth.

## Conclusion

The expanded conceptualisation of irritability proposed here provides a needed framework for understanding how underlying cognitive control processes of proactive and reactive control linked with setting and vulnerability factors through the activation of positive and negative valence systems contributes to outcomes for irritable youth. Based on research spanning clinical and community populations of irritability in childhood, findings point to differential recruitment of proactive and reactive control in the context of negative and positive system activation.

In particular, variations in employing proactive and reactive control appear to arise resulting from interactions between early vulnerability factors and setting factors, including internal mood state (Deveney et al., 2013; He et al., 2013), environmental demands (Adleman et al., 2011; Liuzzi et al., 2020; Toohey & DiGiuseppe, 2017), and expectancies (Dickstein et al., 2007; Proulx et al., 2017) that can lead to system activations (positive or negative). Given the influence of these setting conditions, irritable youth differentially recruit proactive and reactive control when working towards a potential reward, in conditions of nonreward, or in responding to potential threats. Further, increased cognitive load in monitoring environmental distractions and threats and reducing socially unacceptable behavioural responses in irritable youth likely increases more tonic irritability and reduced capacity to effectively employ proactive control in order to meet task goals. At the same time, reduced flexible recruitment of proactive and reactive control as required likely leads to outbursts of frustration (phasic irritability) when presented with negative feedback or the loss of potential or earned rewards due to poorer performance on tasks. Developmentally, irritability in combination with continued inflexible recruitment of cognitive control processes that may be incongruent with the environmental demands, likely contributes to poorer outcomes in youth, such as psychopathology (Kessel et al., 2016). All told, this conceptualisation offers a guiding framework for the investigation of irritability in children and highlights key avenues for treatment research.
